# Supplementary material for: Effectiveness and Safety of Ayurvedic Medicines in Type 2 Diabetes Mellitus Management: A Systematic Review and Meta-Analysis
Source: Front Pharmacol. 2022 Jun 8;13:821810. doi: 10.3389/fphar.2022.821810 (PMC9213670; doi:10.3389/fphar.2022.821810)
Supplement: Supplementary file 1 [file DataSheet1.zip › Supplementary Material/Forest and Funnel Plots/Tinospora cordifolia/LDL-C.pdf]

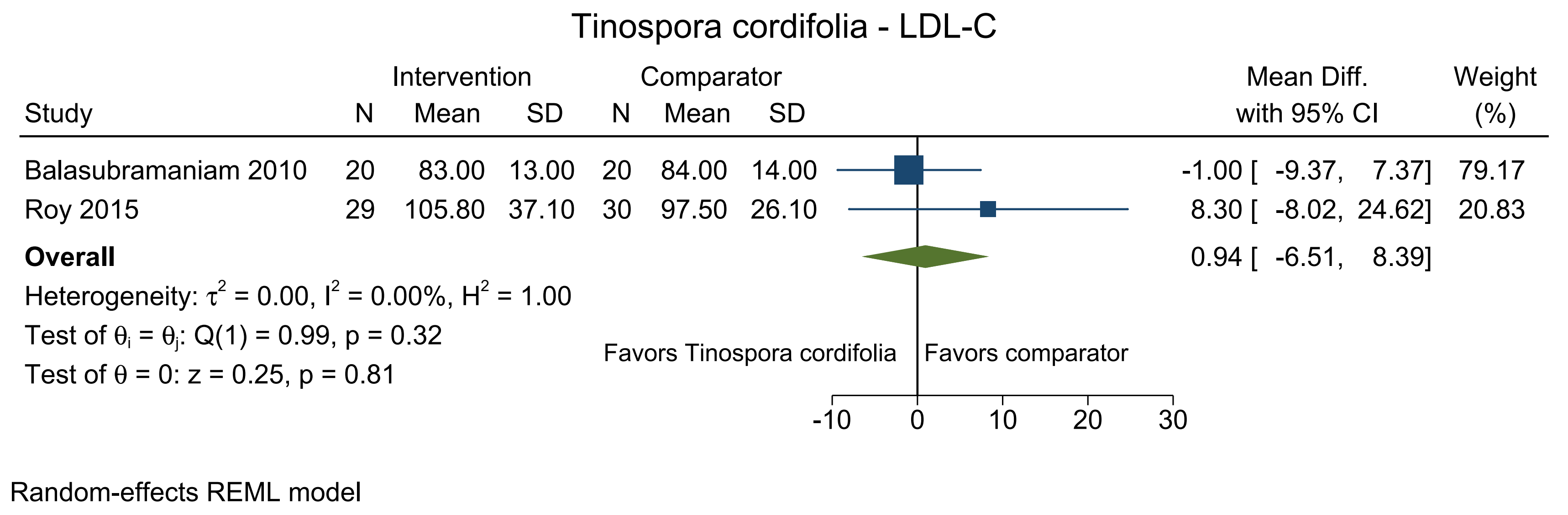
Tinospora cordifolia - LDL-C
Intervention
Comparator
Study
N
Mean
SD
N
Mean
SD
Mean Diff. with 95% CI
Weight (%)
Balasubramaniam 2010 20 83.00 13.00 20 84.00 14.00 -1.00 [-9.37, 7.37] 79.17
Roy 2015 29 105.80 37.10 30 97.50 26.10 8.30 [-8.02, 24.62] 20.83
Overall 0.94 [-6.51, 8.39]
Heterogeneity: τ² = 0.00, I² = 0.00%, H² = 1.00
Test of θi = θj: Q(1) = 0.99, p = 0.32
Test of θ = 0: z = 0.25, p = 0.81
Favors Tinospora cordifolia
Favors comparator
-10
0
10
20
30
Random-effects REML model
